# Effect of Anti-Rheumatic Drugs on Cardiovascular Disease Events in Rheumatoid Arthritis

**DOI:** 10.3389/fcvm.2021.812631

**Published:** 2022-02-03

**Authors:** Yang Baoqi, Ma Dan, Zhao Xingxing, Zhu Xueqing, Wang Yajing, Xu Ke, Zhang Liyun

**Affiliations:** ^1^Third Hospital of Shanxi Medical University, Shanxi Bethune Hospital Shanxi Academy of Medical Sciences, Taiyuan, China; ^2^Tongji Shanxi Hospital, Taiyuan, China; ^3^School of Basic Medicine, Shanxi University of Chinese Medicine, Jinzhong, China

**Keywords:** rheumatoid arthritis, cardiovascular disease, non-steroidal antiinflammatory drugs, glucocorticoids, conventional DMARDs, biological DMARDs, targeted synthesis DMARDs, botanical drugs

## Abstract

Rheumatoid arthritis (RA) is an autoimmune disease characterized by erosive arthritis, which can involve multiple systems. Patients with RA may have a variety of comorbidities, including cardiovascular disease (CVD), lung cancer, lymphoma, infection, osteoporosis, fatigue, depression, colon cancer, breast cancer, prostate cancer, and Alzheimer's disease. Among these comorbidities, the incidence of CVD, lung cancer, lymphoma, infection, and osteoporosis is higher. CVD is a serious complication of RA. The risk of CVD and associated mortality rate in patients with RA is high, and the treatment rate is low. In addition to traditional risk factors, such as age, sex, blood pressure, and diabetes, RA is also associated with inflammation. Furthermore, therapeutic drugs for RA, including non-steroidal anti-inflammatory drugs, glucocorticoids, and disease-modifying anti-rheumatic drugs, have beneficial or harmful effects on cardiovascular events in patients with RA. This article discusses the effects of therapeutic drugs for RA on cardiovascular events.

## Introduction

Rheumatoid arthritis (RA) is a chronic autoimmune disease caused by many factors, which mainly involve inflammatory synovitis and multiple systems. RA pathogenesis involves complex interactions among dendritic cells, macrophages, T cells, B cells, neutrophils, fibroblasts, and osteoclasts (1).

Cardiovascular disease (CVD) is the most common comorbidity of RA, along with atherosclerotic heart disease and heart failure, and CVD is the leading cause of death in patients with RA. A meta-analysis of 14 studies involving 41,490 patients with RA revealed that the risk of CVD increases by 48% and the risk of myocardial infarction (MI) increases by 68% (2). Another meta-analysis revealed that the risk of death by CVD increases by 50% (3). Risk factors of RA associated with CVD include traditional risk factors, such as age, sex, smoking, blood pressure, lipid metabolism, and diabetes, inflammatory immune factors, genetic factors, and drug. All these factors resulting in cardiovascular risk in RA are not independent; they form a network composed of multiple interactive factors, in which bidirectional and synergistic effects can occur.

Atherosclerosis is a chronic inflammatory disease similar to RA, and the two diseases exhibit significant similarities in terms of pathogenesis and genetic determinants. Inflammatory mediators produced by the synovium can alter insulin resistance and lipid distribution or blood pressure either directly by destroying the vascular endothelial cells or indirectly by affecting other tissues (such as the skeletal muscle, liver, or adipose tissues) thereby promoting the progression of atherosclerosis. In addition, inflammation is thought to interfere with the vascular repair. Therefore, inflammation in RA accelerates the development of vascular injury, which leads to the progression of atherosclerosis (4, 5).

**Figure 1:**
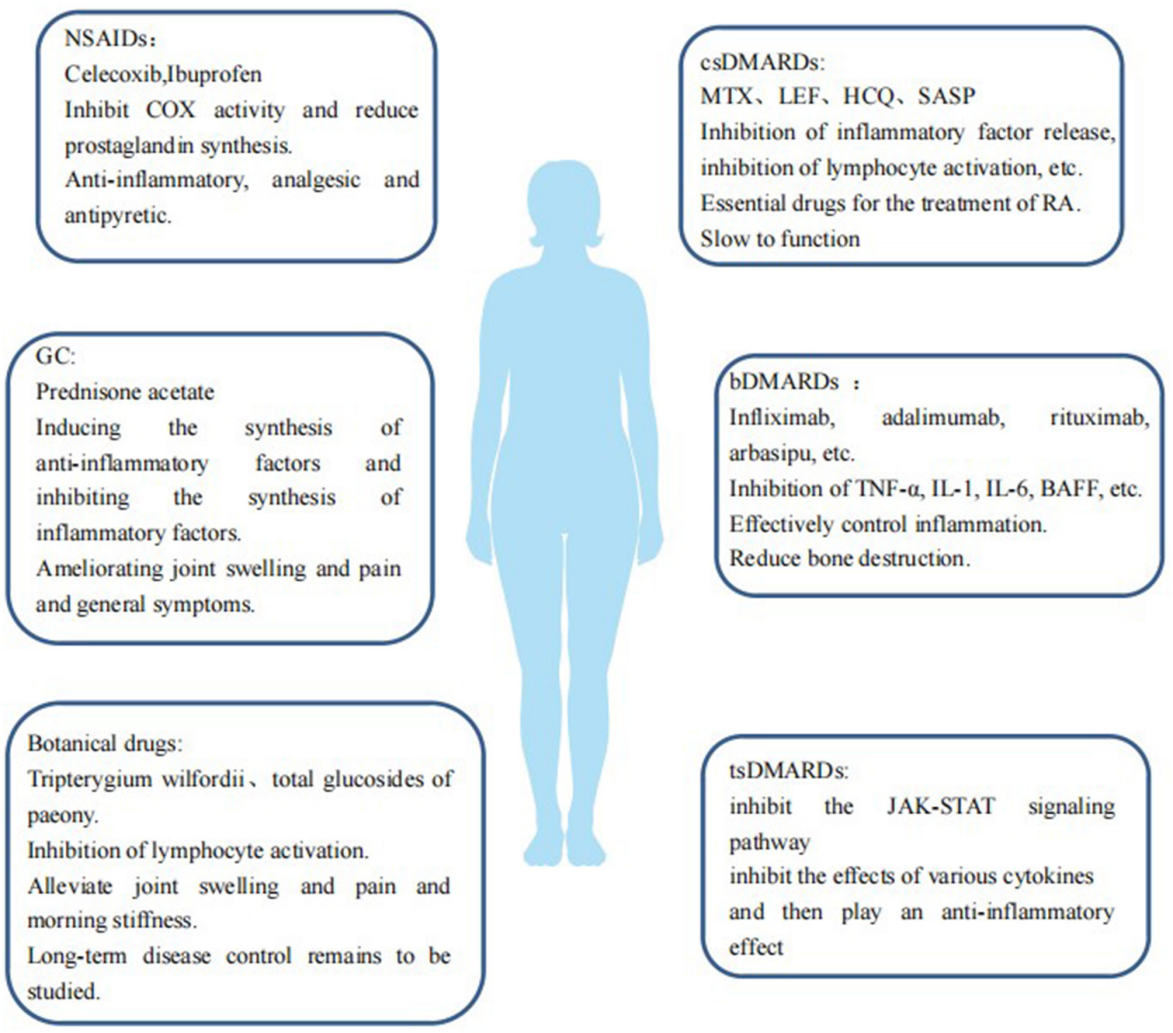
Therapeutic drugs for RA are a double-edged sword in case of RA-induced CVDs. Some drugs may reduce the incidence of CVDs in patients with RA by inhibiting inflammation and improving endothelial function and insulin resistance, and some drugs may impair the mechanism of vascular repair, have harmful effects on the cardiovascular system, and increase the risk of developing CVDs. We will explain the effects of these drugs on the cardiovascular risk of patients with RA.

Drugs for RA mainly include non-steroidal anti-inflammatory drugs (NSAIDs), glucocorticoids (GC), disease-modifying anti-rheumatic drugs (DMARDs), and botanicals (Figure 1). (1) NSAIDs such as celecoxib and ibuprofen exert anti-inflammatory, analgesic, and antipyretic effects by inhibiting cyclooxygenase (COX) activity and reducing prostaglandin synthesis. (2) GCs, such as prednisone acetate, can induce the synthesis of anti-inflammatory factors, inhibit the synthesis of inflammatory factors, inhibit the expansion of capillary vessels, and reduce edema and exudation of inflammatory fluids, thus playing an anti-inflammatory role, quickly improving joint swelling and pain and general symptoms. (3) DMARDs should be used as early as possible once RA diagnosis is established as they can delay and control the progression of the disease, but DMARDs have no obvious analgesic and anti-inflammatory effects. 3.1 Conventional DMARDs (csDMARDs) take effect slowly over 4–12 weeks; therefore they are called slow-acting anti-rheumatic drugs. csDMARDs mainly include methotrexate (MTX), leflunomide (LEF), hydroxychloroquine (HCQ), sulfasalazine (SASP), cyclosporine (CsA), and azathioprine (AZA). 3.2 Biological DMARDs (bDMARDs) are important drugs that actively and effectively control inflammation, which can reduce bone destruction, hormone consumption, and osteoporosis. bDMARDs include tumor necrosis factor (TNF)-α antagonist, interleukin (IL)-1 antagonist, IL-6 antagonist, anti-CD20 monoclonal antibody, and T cell co-stimulation signal inhibitor. 3.3 Targeted synthesis DMARDs (tsDMARDs), including JAK inhibitors (tofacitib, baritinib, upatinib), inhibit the JAK-STAT signaling pathway, as well as the effects of various cytokines, and play an anti-inflammatory role (6). 4. Currently, in China *Tripterygium wilfordii* and total glucosides of paeony (TGP) are commonly used as botanical drugs, some of which have a good effect on relieving joint swelling and pain and morning stiffness, but the effects of long-term disease control warrant further study.

## NSAIDs

NSAIDs are a class of drugs that play anti-inflammatory, analgesic, and antipyretic roles by inhibiting COX and blocking the conversion of arachidonic acid (Aa) to prostaglandin (PG). COX plays an important role in regulating cardiovascular homeostasis, and it functions by regulating the ratio of PGI2 to TXA2 (7). The unbalanced TXA2/PGI2 ratio changes cardiovascular homeostasis and leads to various cardiovascular complications. PGI2 is mainly controlled by COX-2, which is found in the endothelial cells of large blood vessels and inhibits platelet aggregation, relaxing blood vessels, and resisting smooth muscle proliferation. TXA2 is mainly controlled by COX-1, which is found in platelets and can cause platelet aggregation, vasoconstriction, and smooth muscle thickening. Some studies suggest that PGI2 plays an important role in limiting the cardiovascular effects of TXA2. Elevated blood pressure caused by TXA2 can accelerate the formation of atherosclerosis and directly lead to vascular reconstruction. The vascular structure eventually changes with the increase in COX-2 inhibition time. Inhibition of COX-2 may also cause unstable atherosclerotic plaques, leading to thrombosis.

The treatment of RA with NSAIDs mainly exerts anti-inflammatory and analgesic effects by inhibiting COX and blocking Aa from transforming into PGs, which ameliorates the symptoms of RA, but these drugs cannot prevent its progression. NSAIDs can be divided into non-selective NSAIDs, selective COX-1 inhibitors, and selective COX-2 inhibitors (such as rofecoxib and celecoxib).

Non-selective COX inhibitors are traditionally considered to have cardiovascular protective effects. However, recent studies have found that these drugs also have potential cardiovascular risks, and their cardiovascular risks depend on the degree of inhibition of COX-1 and COX-2 the smaller the degree of COX-1 inhibition, the greater the degree of COX-2 inhibition, and the greater the cardiovascular risk. According to a meta-analysis previously conducted, the relative risks of CVD in patients receiving COX-2 inhibitors or non-selective NSAIDs were 1.36 (95% confidence interval [CI] 1.10–1.67), and 1.08 (95%CI:0.94–1.24), respectively, while the relative risks of MI, stroke, and major cardiovascular events were 1.13 (95%CI:0.93–1.37), 2.15 (95%CI:1.19–3.87), and 1.56 (95%CI:0.82–2.97), respectively. This finding indicates that all NSAIDs increase the risk of CVD (8). In addition, non-selective NSAIDs not only increase the blood pressure of healthy and hypertensive people but also interfere with antihypertensive drugs other than calcium channel blockers (9).

Low-dose selective COX-1 inhibitors, such as aspirin have cardiovascular protective effects because they can prevent thrombosis and are used for secondary prevention of CVD. However, taking a large dose of these drugs for a long time can inhibit thrombin synthesis and increase bleeding tendency (7). A study showed that naproxen and flurbiprofen have significant antiplatelet effects when used at their usual doses. In addition, the antiplatelet effect of aspirin is decreased significantly when aspirin is taken after ibuprofen or mefenamic acid. Other NSAIDs do not have a significant effect on the antiplatelet effect of aspirin (10). Taking aspirin and naproxen simultaneously increases the recurrence rate of MI, with a 30-day risk ratio of 1.13 and a 60-day risk ratio of 1.83, compared to taking aspirin alone (11).

Studies have shown that selective COX-2 inhibitors inhibit the synthesis of prostacyclin in endothelial cells by selectively inhibiting COX-2, which destroys the balance between TXA2 and PGI2, leading to atherosclerosis, thrombosis, and other cardiovascular complications (9). Both high- and low-dose rofecoxib can lead to cardiovascular events, which are dose-dependent, in the early stages of RA treatment. A case-control study showed that both high-dose (> 25 mg/day) and low-dose (≤25 mg/day) rofecoxib administration can lead to cardiovascular events in the early stage of drug administration (≤4 months), and the cardiovascular risk of high-dose rofecoxib treatment is higher than that of low-dose rofecoxib treatment (12). Rofeixib (50 mg/day) increased the incidence of MI by 0.5% in ~8,000 patients with RA, while the incidence of MI increased by 0.1% in patients treated with naproxen (500 mg twice a day). Rofeixib is associated with thrombosis compared with naproxen (13, 14). A network meta-analysis of seven NSAIDs (naproxen, ibuprofen, diclofenac, celecoxib, etocoxib, rofecoxib, and romeixib) showed that, compared with placebos, rofecoxib treatment had the highest risk of MI, etocoxib had the highest risk of vascular death, ibuprofen had the highest risk of stroke, and naproxen had a lower risk of CVD events (15).

As aforementioned, the cardiovascular risks of NSAIDs depend on the inhibition degree of COX-1 and COX-2. Therefore, NSAIDs should be carefully selected for cardiovascular high-risk groups, and if necessary, drugs with a low COX-2 inhibition effect, such as naproxen and ibuprofen, should be selected as far as possible.

## GCs

Although the exact mechanism of RA is unclear, Th cell subsets play an important role in its pathogenesis. CD4+T cells, especially Th1 and Th17 cells, and Th1 cells activate macrophages and increase their ability to produce TNF. In addition, IL-12, IL-18, and IFN-γ, which are the drivers of Th1 differentiation, were also found in the synovial tissues of patients with RA. GCs are lipophilic substances that can easily pass through the cell membrane, bind to the GC receptor in the cytoplasm, and reduce IFN-γ production by T cells by inducing apoptosis of Th1 cells (16), preventing phospholipid release and reducing eosinophil activity, thus inhibiting the systemic inflammatory response (17). Thus, GCs play a very important role in the treatment of RA as they can quickly inhibit inflammatory activity.

GCs can quickly and effectively inhibit inflammation associated with RA, but their use is still controversial due to various related cardiovascular adverse reactions, including hypertension (which is common in patients receiving ≥7.5 mg prednisone daily for ≥6 months), dyslipidemia, insulin resistance, and type 2 diabetes (18, 19).

Using the minimum effective dose of GC may reduce the increase in CV risk caused by inflammation by controlling the inflammatory process; as GCs may increase the risk of CV by causing changes in blood lipid levels, insulin resistance, diabetes, hypertension, and obesity with an increase in their exposure time and cumulative dose. Results of a previous study showed that the CVD risk ratio in patients who receive GC treatment with prednisone at 1–7 mg/day was 1.78, and that in those who received prednisone at ≥7.5 mg/day was 2.61 (20), In addition, patients who received high-dose GC (> 7.5 mg/day prednisone) had twice the risk of heart disease compared with patients who did not receive GC treatment (21). A multivariate analysis study showed that the risk of MI increased by 68% in patients with RA treated with GC, and single-factor analysis showed that daily dose, cumulative course of treatment, and total cumulative dose were significantly correlated with an increase in MI risk (22). The minimum effective dose is recommended in a dose- and duration-dependent manner for patients receiving corticosteroids, considering their correlation with increased CVD risk (23, 24).

Therefore, it is suggested that the minimum effective dose should be used as much as possible and the course of treatment should be considered for patients who need GC treatment. Additionally, the blood pressure, blood lipid levels, and blood sugar levels of the patient should be closely monitored, and other drugs should be administered if necessary to antagonize their side effects.

## Conventional DMARDs

### MTX

MTX is an immunosuppressive drug and a folic acid analog, which competitively inhibits dihydrofolate reductase and prevents dihydrofolate (FH2) from being converted into tetrahydrofolate (FH4). FH4, a carrier of a one-carbon unit, participates in purine and pyrimidine metabolism, and inhibition of FH4 synthesis leads to the damage of purine and pyrimidine metabolism, and the inhibition of amino acid and polyamine synthesis, which leads to the production and release of adenosine, showing the most direct anti-inflammatory effect. According to reports, MTX inhibits the binding of IL-1β and IL-1βR and prevents IL-1β-induced inflammatory reactions (1, 25).

MTX can significantly reduce CVD mortality and delay the risk by 3–4 years in patients with RA (26), and the relative risk (RR) (0.81) of MI can be significantly reduced via MTX treatment (27). MTX plays an anti-inflammatory role by inhibiting the production of TNF-α, IL-1, and IL-8 by monocytes, enhancing the synthesis of IL-1R antagonist protein and the expression of soluble TNF receptors. However, some studies have shown that MTX does not only affect the inflammatory reaction but also upregulates the concentration of homocysteine in patients with RA, which may promote atherosclerosis in patients with RA who already suffer from atherosclerotic heart disease (28). Studies have shown that the continuous use of MTX in patients with RA within 1 year of onset can reduce the risk of CVD by 20%; however, a cumulative dose of MTX cannot significantly reduce the risk of CVD with the prolongation of the disease course, which may be related to the improvement in homocysteine levels and decreased CVD protection upon long-term use of MTX (29–31). MTX can prevent intimamedia thickening, as MTX is associated with lower carotid intima-media thickness (CIMT) and femoral artery intima-media thickness (FIMT) it was found that the CIMT and FIMT of patients administered with MTX at ≥20 mg/week were significantly reduced compared to those administered different MTX doses (32). In addition, MTX can improve endothelial function (33) and lipid status by increasing the HDL levels (34), thus inhibiting atherosclerosis. Therefore, a large number of studies have shown that MTX has a positive effect on the inhibition of atherosclerosis in patients with RA.

Therefore, MTX has an inhibitory effect on atherosclerosis in patients with RA, but folic acid should be properly supplemented during MTX administration as MTX can increase homocysteine concentration in RA patients.

### SASP

SASP is an anti-cytokine drug that affects the absorption and metabolism of folic acid, inhibits the chemotaxis of leukocytes and the activity of proteolytic enzymes, and its potential NF-κB and TNF-α inhibitory activities can further control the symptoms of patients with RA (35, 36). SASP may help ameliorate coronary heart disease by improving the blood lipid levels and controlling endothelial dysfunction and carotid artery remodeling induced by inflammation (37, 38). In addition, SASP may have beneficial effects on the cardiovascular outcomes of RA by inhibiting platelet aggregation induced by arachidonic acid (39).

At present, studies have shown that SASP has beneficial effects on CVD due to RA, and no reports of CVD have been found.

### HCQ

HCQ is an antimalarial drug that interferes with lysosomal activity and autophagy. Lysosomes are involved in the recovery of the cell matrix, antigen processing, and major histocompatibility complex class II molecule presentation, which indirectly promotes immune activation (40). The decrease in lysosomal activity can inhibit the function of lymphocytes and has immunoregulatory and anti-inflammatory effects. Autophagy is also involved in antigen expression and immune activation (41). HCQ can indirectly lower expression of the anti-inflammatory cytokines in different cell types. HCQ inhibits the production of IL-1, IL-6, TNF-α, and IFN-γ by monocytesin *in vitro*. In addition, HCQ treatment inhibits the production of TNF-α, IFN-α, IL-6, and CCl4 (also known as MIP1β) in plasmacytoid dendritic and natural killer cell cocultures stimulated by the RNA-containing immune complex (42), thereby reducing inflammation.

HCQ is widely considered to confer vascular protection. Studies have shown that the CVD risk of patients with RA decreases by 72% upon HCQ administration (43). HCQ can inhibit platelet aggregation and erythrocyte aggregation, prevent thrombosis (44), improve insulin sensitivity, lower blood sugar levels (45), and reduce the levels of total cholesterol and low-density lipoprotein (46), thereby reducing the risk of CVD (47). However, some studies have shown that taking HCQ for an extended time can cause serious heart diseases, as well as sodium and calcium channel blockages, affecting membrane stability and resulting in atrioventricular blockages, widening the QRS interval, and prolonging the QT interval, causing conduction disorder (48). However, these serious side effects are rare.

At present, most studies report that HCQ might confer vascular protection, and some studies show that the long-term use of HCQ can cause serious heart diseases, but these effects are rare. Therefore, electrocardiograms should be used to monitor the patients administered HCQ, and HCQ administration should be terminated if there is an abnormality.

### LEF

LEF is an isoxazole immunomodulator with antiproliferative activities, which can inhibit the activities of tyrosine kinase and lactate dehydrogenase and interfere with the synthesis of pyrimidine, which is necessary for lymphocyte activation. Pyrimidine synthesis obstruction can significantly inhibit the activation and proliferation of T cells, effectively inhibit cellular immunity, and control the development of RA-related diseases (36). The activity of LEF *in vivo* is mainly due to its active metabolite A77-1726, which inhibits the activity of dihydroorotate dehydrogenase (49), playing a key role in the *de novo* synthesis of pyrimidine ribonucleotide uridine monophosphate. This inhibition prevents the transition from the G1 to the S phase and lymphocyte cloning and expansion, thereby relieving symptoms and delaying the progression of RA (50). In addition, LEF shows anti-inflammatory effects by increasing the synthesis of immunosuppressive cytokines, such as TGF-β1 (51).

Studies have shown that A77-1726 treatment can alleviate myocardial hypertrophy induced by pressure overload or angiotensin II *in vivo* and myocardial hypertrophy induced by agonists. In addition, the administration of A77-1726 prevents the induction of cardiac fibrosis by inhibiting the transformation of cardiac fibroblasts into muscle fibroblasts. Studies have also shown that the protective effect of A77-1726 does not depend on T lymphocyte inhibition, but on inhibiting the activation of the protein kinase B signaling pathway (52). However, LEF has the risk of increasing blood pressure, which should be considered (53).

Collectively, LEF can alleviate myocardial hypertrophy induced by various factors and prevent cardiac fibrosis, but it may cause hypertension and its administration requires paying attention to monitoring of the patient's blood pressure during use.

### CsA

CsA inhibits the response of activated T cells to IL-2 *in vitro* (54). Furthermore, CsA can selectively inhibit the transcription of IL-2 in T cells, inhibit the synthesis and secretion of IFN-γ and IL-3, and promote the apoptosis of macrophages, thus inhibiting the secretion of inflammatory factors and alleviating joint damage (55). CsA can inhibit the transcription of IL-17mRNA and the secretion of IL-17 from CD4+T lymphocytes in patients with RA, thereby reducing the inflammatory response to RA and significantly enhancing bone tissue destruction (56). Patients treated with CsA had lower intima-media thickness and plaque prevalence (32). CsA also prevented myocardial hypertrophy by inhibiting Ca^2+^/calmodulin-dependent serine/threonine protein phosphatase (57). However, the use of CsA can lead to an increase in blood pressure, the mechanism for which is not completely clear. The dose-related increase in the blood pressure during CsA treatment is statistically significant. Taking 1–4 mg/kg every day is related to a 5 mmHg increase in blood pressure, and taking > 10 mg/kg every day is related to an 11 mmHg increase in blood pressure. Therefore, the blood pressure should be assessed and cardiovascular risk factors should be monitored frequently before initiating CsA therapy (58, 59). Moreover, there is some evidence that CsA treatment can promote, in part, endothelial dysfunction by increasing the production of oxidative stress -related substances (60). Treatment with CsA is also associated with microvascular injury, which promotes endothelial injury (61).

Collectively, CsA reduces intima-media thickness and plaque prevalence and prevents the occurrence of myocardial hypertrophy, but can also lead to an increase in blood pressure and promote endothelial injury. Therefore, the risk factors for CVD should be evaluated and monitored upon CsA administration.

### AZA

AZA is a non-specific immunosuppressive agent formed by substituting methyl imidazole for hydrogen and sulfur atoms in 6 mercaptopurine (6-MP). AZA can inhibit the synthesis of purines by interfering with the purine metabolism, and further inhibit the synthesis of DNA, RNA, and protein, and the proliferation of lymphocytes to exert immunosuppressive effects (62). Studies have shown that AZA pretreatment can inhibit excessive oxidative stress and inflammation caused by myocardial ischemiareperfusion by downregulating the TLR4 signaling pathway, promoting oxidation-antioxidation balance, and alleviating myocardial injury caused by ischemia-reperfusion to a certain extent (63).

## Biological DMARDs

### TNF Antagonists

TNF regulates leukocyte activation and maturation, cytokine and chemokine release, and reactive oxygen species (ROS) production. Therefore, TNF is the central regulator of the inflammatory cascade to initiate and amplify inflammation. TNF activates endothelial cells, induces expression of adhesion molecules and receptors of pro-inflammatory cytokines and chemokines, and promotes the synthesis and release of various inflammatory cytokines and chemokines, thus supporting the recruitment of activated leukocytes to inflammatory lesions. TNF may promote the inflammatory cascade in the artery wall during the development of atherosclerosis, partly by promoting endothelial cell injury (64). TNF also promotes the apoptosis of endothelial cells and inhibits the activity of endothelial progenitor cells that maintain endothelial repair (65). TNF additionally promotes endothelial injury by recruiting immune cells, such as neutrophils, that mediate tissue destruction (66). Furthermore, TNF promotes oxidative stress, which can directly damage the bioavailability of NO, resulting in endothelial dysfunction.

TNF antagonists are divided into monoclonal antibodies and receptor-antibody fusion proteins. Monoclonal antibodies include infliximab and adalimumab. Receptor-antibody fusion proteins include the all-human soluble anti-TNF fusion protein etanercept and recombinant human type II TNF receptor-antibody fusion proteins rhTNFR-Fc.

Studies have reported that serum leptin and adiponectin concentrations increase and insulin sensitivity and endothelial function improve after infliximab treatment (67, 68), which affect inflammation burden and plaque vulnerability and reduce the risk of cardiovascular disease (69, 70).

Studies have shown that adalimumab can significantly ameliorate dyslipidemia, endothelial dysfunction, carotid atherosclerosis, and arterial stiffness in patients with RA, thereby reducing the risk of CVD (71).

According to a study, etanercept can reduce pulmonary artery pressure in a rat model of monocrotaline-induced pulmonary hypertension through inhibiting the secretion of inflammatory factors by macrophages, in addition to reducing the degree of vascular inflammation and vascular wall thickening in pulmonary arterioles (72). Studies have shown that etanercept can effectively reduce myocardial injury caused by myocardial ischemia-reperfusion after trauma and exhibits a robust protective effect on the injured myocardium, the mechanism for which may be related to the activation of the Notch1 signaling pathway. However, due to the lack of samples in the aforementioned study, the relationship between etanercept treatment and long-term mortality of myocardial ischemia-reperfusion injury requires further in-depth study (73).

rhTNFR-Fc can protect myocardial cells, reduce the release of TNF from myocardial cells, maintain the integrity of the membrane structure, inhibit the leakage of intracellular troponin, improve the prognosis associated with reperfusion-injured myocardium and protect the myocardium. A study reported that after treatment with rhTNFR-Fc, the serum homocysteine levels decrease; further, the increase in coronary blood flow reserve and brachial artery endothelium-dependent vasodilation rate delay the progression of coronary heart disease and reduces the occurrence of CVDs (74).

A meta-analysis showed that the use of TNF antagonists in patients with RA can reduce all CVD risks, with an RR of 0.46, for which the MI RR was reported as 0.81 (75). In summary, TNF inhibitors exhibit a variety of myocardial protective mechanisms, including promoting cholesterol transport, improving glucose metabolism, downregulating adhesion molecules, and resisting the effects of inflammation on blood coagulation.

Long-term overstimulation of TNF leads to an impaired ability of the β-adrenergic receptor (β-AR) to respond to natural agonists, resulting in systolic dysfunction, cardiac hypertrophy, and induction of cardiomyocyte apoptosis in patients with heart failure (76). Moreover, chronic stimulation of TNF can increase the formation of other pro-inflammatory cytokines, such as IL-6 and IL-1, which are also involved in the pathogenesis of Congestive Hearts Failure (CHF) (77). As far as heart failure is concerned, TNF inhibitor treatment can protect the heart by controlling inflammation. A study reported that anti-TNF treatment does not increase the risk of heart failure (78). However, under some pathophysiological conditions, TNF may play a protective role, limit infarct size, and provide immunomodulatory activity in the state of cardiac dysfunction. It should be noted that low levels of TNF are necessary to protect the myocardium from injury, while high systemic TNF levels lead to the development of ventricular dysfunction. Another study reported that etanercept and infliximab (5 and 10 mg/kg) do not improve cardiac function; therefore, anti-TNF drugs are not recommended for patients with heart failure (79).

To sum up, TNF antagonists can protect the myocardium by promoting cholesterol transport, improving glucose metabolism, downregulating adhesion molecules, and resisting the influence of inflammation on blood coagulation, but they are not recommended for patients with heart failure.

### Anti-CD20 Antibody (Rituximab)

B1 cells mainly resist atherosclerosis by producing natural IgM antibodies, and B2 cells (including follicles and marginal B cells) are considered to promote atherosclerosis (80). Anti-CD20 treatment consumed B2 cells, whereas B1 cells remain intact.

Studies have shown that anti-CD20-mediated B cell depletion can reduce plaque burden in mice susceptible to atherosclerosis. In addition, in a MI mouse model, CCL7(MCP-3) derived from B cells induced monocyte mobilization, which led to increased tissue damage. Treatment with anti-CD20 can lead to the depletion of B cells, decreased CCL7 derived from B cells, decreased infarct area, and improved cardiac remodeling. Therefore, it can be speculated that the prognosis of MI in patients treated with rituximab may be beneficial (81). Rituximab is generally well-tolerated, although some patients (<10%) may develop peripheral edema, hypertension, hypotension, or arrhythmia after treatment. In rare cases, acute MI may occur during or shortly after the use of rituximab. The potential mechanism underlying the manifestation of these adverse reactions is unclear, but in acute transfusion reactions, the levels of several pro-inflammatory cytokines (such as IL-6, IL-8, and TNF) may increase, which may lead to coronary artery contraction, platelet activation, or plaque rupture. Therefore, patients with symptoms of ischemic heart disease should be made aware of the aforementioned complications (82).

### Regulator of the Costimulatory Signal of T Cell Activation (Abatacept)

Abatacept has a short-term effect on endothelial function. The short-term improvement of microvascular endothelial function on abatacept administration coincides with the gradual deterioration of vascular endothelial function (83), a phenomenon that can induce a shift in the polarization of adipose tissue macrophages from the pro-inflammatory M1 phenotype to the anti-inflammatory M2 phenotype (84), increase the proliferation and inhibitory activity of Treg cells (85), improve insulin resistance, increase insulin sensitivity index, and reduce the risk of CVD (86). Compared with TNF inhibitors, the risk of using abatacept combined with CVD in patients with basic diseases is reduced by 20%, which indicates that abatacept has higher CVD safety than TNF inhibitors, especially for patients with RA who are older and have CVD (87).

### IL-6 Inhibitor (Tocilizumab)

Tocilizumab targets both soluble IL-6R and membranebound IL-6R, thus preventing IL-6 from interacting with IL-6R, inhibiting the activation of T and B cells, and controlling inflammatory reactions (88, 89); it can also improve endothelial function (90). Low-dose of tocilizumab inhibits the migration of human aortic smooth muscle cells (HASMCs) but does not affect their proliferation, and significantly inhibits the expression of intercellular adhesion molecule (ICAM-1) and matrix metalloproteinase-9 in HASMCs induced by high glucose, while high-dose tocilizumab can only inhibit the expression of ICAM-1, thus playing an anti-atherosclerotic role (91). To sum up, IL-6 inhibitors have atherosclerosis inhibiting effects.

### IL-1 Receptor Antagonist (Anakinra)

IL-1 is the main cytokine involved in local and systemic inflammation, and blocking the activity of IL-1 can alleviate inflammatory reactions and block the progression of RA. The expression of vascular endothelial cell markers (such as endothelin-1) changed nitric oxide stress was relieved, and left ventricular function was improved after treatment with anakinra in patients with RA and coronary artery disease, and the coronary blood flow reserve and myocardial deformation were also improved (92–94). Studies have shown that anakinra can alleviate cardiac remodeling after MI without increasing the risk of heart failure (95, 96), and anakinra also restores left ventricular diastolic function in patients with existing heart failure, especially in patients with diastolic heart failure (92). Anakinra is therefore suitable for patients with MI, as it improves cardiac remodeling after infarction without increasing the risk of heart failure.

## Targeted Synthesis DMARDs

### JAK Inhibitor (Tofacitinib and Baricitinib)

JAK inhibitors target the cytokine signaling pathway involved in the pathogenesis of RA and provide alternative treatment options for RA. Different cytokines transmit activation signals through the JAK signaling pathway and exhibit anti-coagulation and pro-coagulation effects. Blocking one signaling pathway preferentially disturbs the balance between the pro-thrombotic and anti-thrombotic effects. One study showed that patients treated with JAK inhibitors are at an increased risk of developing thromboembolic events. This risk is especially high for patients who are already at a risk of manifesting venous thromboembolic events (VTEs), including a history of CVD, weight gain, hypercoagulable state, cancer, and a previous history of VTE. It is also high for patients receiving estrogen therapy, major surgery, or those with movement disorders (97). An indirect meta-analysis showed that tofacitinib has better VTE safety characteristics than baricitinib (98). A clinical study showed that among patients ≥50 years of age and with ≥1 cardiovascular risk factors, patients who received tofacitinib 10 mg twice a day had a higher incidence of pulmonary embolism than patients who received TNF inhibitors (99). Therefore, patients with risk factors related to thromboembolic events, such as old age, smoking, and history of thromboembolism should be cautious or suspend the use of JAK inhibitors.

## Botanical Drugs

In China, in addition to NSAIDs, GC, and DMARDs, some botanicals are also used to treat RA.

### Tripterygium Wilfordii

*Tripterygium wilfordii* can reduce the expression of TNF-α, IL-6, and IL-1β, regulate the NF-κB signaling pathway, regulate the COX-2/prostaglandin E2 axis of synovial fibroblasts in patients with RA, inhibit Th17 cell differentiation, inhibit the differentiation of B cell into plasma cells, induce the apoptosis, differentiation, and maturation of dendritic cells, induce the apoptosis of macrophages, inhibit macrophage activation, and regulate the Toll-like receptor signaling pathway to activate NKT cells to promote the release of cytokine IFN-γ, play an anti-inflammatory and immune regulatory role, further reduce bone destruction, protect cartilage tissue, and improve circulation (100, 101).

*Tripterygium wilfordii* protects from kidney damage caused by salt-sensitive hypertension by inhibiting inflammatory reactions dominated by mononuclear macrophage infiltration in rats. High doses of triptolide (100 μg/kg) can ameliorate cardiac hemodynamic disturbances, enhance myocardial contractility, and promote the recovery of cardiac function after ischemia-reperfusion in rats. At the same time, it can ameliorate the pathological changes upon ischemia-reperfusion injury and reduce the number of macrophages infiltrating the heart tissue, reduce the infarct area, and alleviate the inflammatory reaction and enhance the antioxidant capacity of ischemia-reperfusion in rats (102). However, *Tripterygium wilfordii* has toxic side effects, which can cause cardiac toxicity. Clinical manifestations of *Tripterygium wilfordii* toxicity include chest tightness, palpitation, and arrhythmia, and in severe cases, insufficient blood supply to the heart, sudden drop in blood pressure, shock, or heart failure, which is a life-threatening event (103–105).

*Tripterygium wilfordii* can ameliorate ischemia-reperfusion injury, and can be used for RA therapy in patients with angina pectoris and MI. However, patients should be warned about the aforementioned side effects of *Tripterygium wilfordii*.

### Total Glucosides of Paeony (TGP)

TGP can inhibit the proliferation and activation of B cells by downregulating the expression of soluble CD23, which is a marker of B lymphocyte activation in the blood of patients with RA. Additionally, TGP can inhibit the activation of the TLR4/NF-κB signaling pathway, reduce the synthesis and release of inflammatory mediators, such as TNF-α, IL-6, and IL-1β, alleviate the inflammation of the joint synovial tissue, inhibit the excessive proliferation of synovial cells, and promote synovial cell apoptosis (106–109).

Studies have shown that TGP pretreatment can effectively inhibit the development of aortic lesions in atherosclerotic rats, increase the activities of superoxide dismutase and catalase, reduce the levels of ROS, reduce the expression of TNF-α, IL-1β, and IL-6, and increase the expression of IL-10, suggesting that TGP can protect atherosclerotic rats by inhibiting oxidative stress injury and inflammatory reactions. A study reported that the degree of atherosclerosis in the TGP group, especially in the 200 mg/kg/day) TGP group, was significantly reduced compared with that in the control group (110). Studies have shown that TGP has a protective effect on ischemia-reperfusion injury in the rat myocardium *in vivo* and *in vitro*, a phenomenon that can improve the hemodynamic parameters and reduce the infarct size. In addition, TGP has an anti-myocardial remodeling effect (111). Studies have shown that insulin sensitivity in insulin-resistant rats was increased, the degree of lipid metabolism disorder was reduced, and the blood pressure was reduced after TGP treatment, which indicates that TGP has a significant positive effect on hyperlipidemia and hypertension in insulin-resistant rats (112). TGP can effectively reduce the levels of plasma total cholesterol, low-density lipoprotein cholesterol, triglycerides, and very-low-density lipoprotein cholesterol, and increase the concentration of high-density lipoprotein cholesterol, thus inhibiting atherosclerosis to some extent (113, 114). Collectively, TGP has a protective effect on CVDs, especially for patients with MI or insulin resistance.

**Figure 2:**
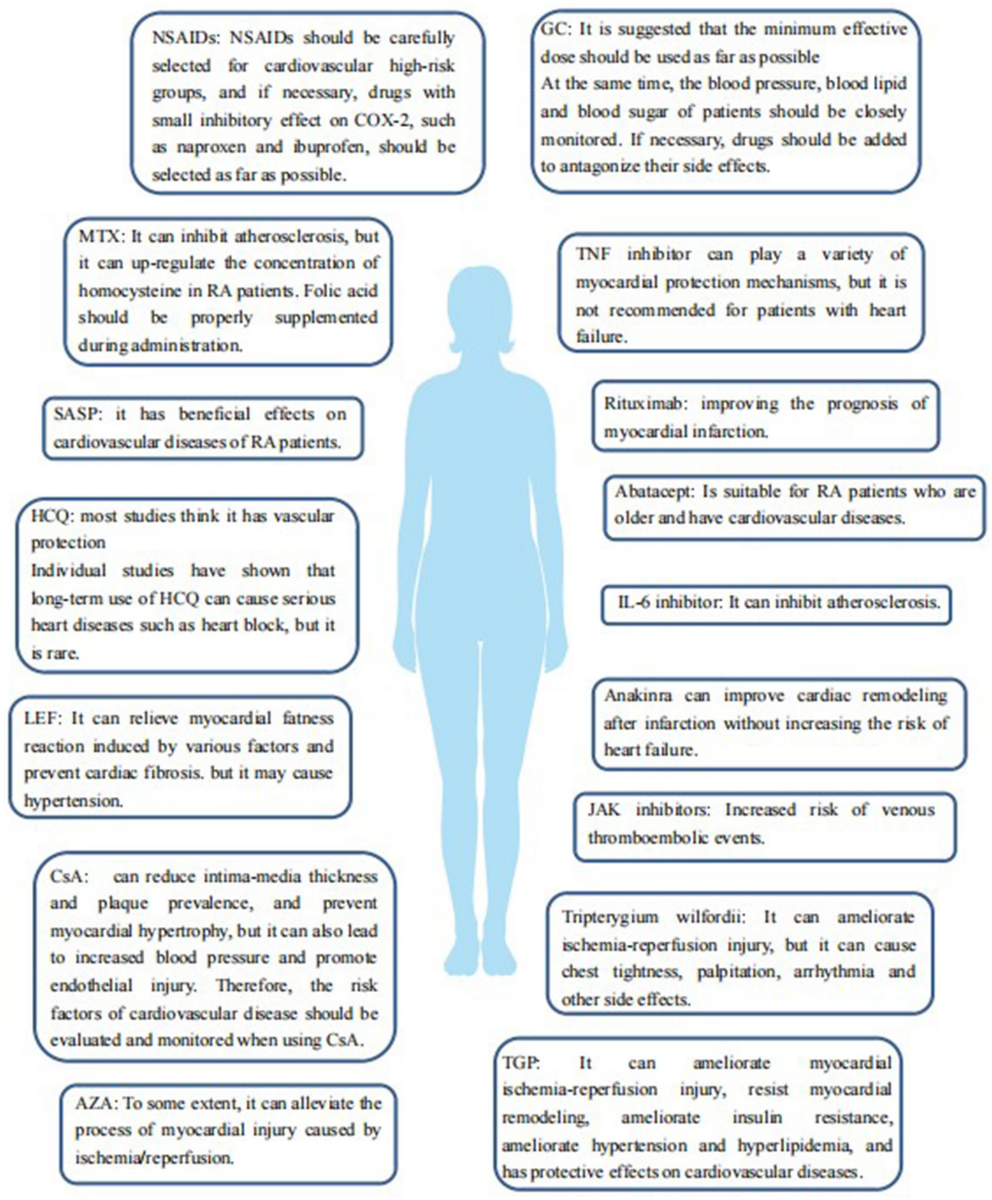
The effect of various drugs for the treatment of RA, including NSAIDs, GCs, DMARDs, and botanicals, on cardiovascular risk in patients with RA.

Figure 2, Table 1 summarize the effects of various drugs for RA, including NSAIDs, GC, DMARDs, and botanical drugs, on the cardiovascular risk of the levels of RA.

**Table 1:** Cardiovascular risks and preventive measures of common antirheumatic drugs.

**Drugs**		**Impact on the cardiovascular risk**	**Suggestion**	**References**
NSAIDs	Non- selectively NSAIDs	Its cardiovascular risk depends on the inhibition degree of COX-1 and COX-2. The smaller the inhibition degree of COX-1, the greater the inhibition degree of COX-2, and the greater its cardiovascular risk may be	People at high cardiovascular risk should use NSAIDs with caution. If necessary, use drugs that have a small inhibitory effect on COX-2, such as naproxen and ibuprofen	(8, 9, 12–15)
	Selective COX-2 inhibitors	Selective inhibition of COX-2 inhibits the synthesis of prostacyclin in endothelial cells, which destroys the balance between TXA2 and PGI2, leading to atherosclerosis, thrombosis, and other cardiovascular complications		
GC		The minimum effective dose of GC may reduce the increase of CV risk caused by inflammation by controlling the inflammatory process Increasing exposure time and cumulative dose may increase the risk of cardiovascular disease through changes in blood lipid levels, insulin resistance or diabetes, hypertension, obesity, etc.	Use the smallest effective dose as much as possible and consider the course of treatment. At the same time, closely monitor the patient's blood pressure, blood lipids, blood sugar, etc., and add drugs when necessary to antagonize its side effects.	(18–24)
csDMARDs	MTX	It can inhibit atherosclerosis by anti-inflammatory, improving endothelial function, preventing intima-media thickening, and improving lipid status. However, with the prolongation of the course of disease, the CVD protective effect of MTX may be weakened because MTX increases homocysteine level	Folic acid should be supplemented appropriately during medication	(26–34)
	SASP	By improving blood lipid level, controlling endothelial dysfunction and carotid artery remodeling induced by inflammation may help to ameliorate coronary heart disease and inhibit platelet aggregation mediated by arachidonic acid.	–	(37–39)
	HCQ	HCQ can reduce the risk of cardiovascular disease by preventing thrombosis, lowering blood sugar, and improving lipid status However, some studies have shown that long-term use of HCQ can cause sodium and calcium channel block, which leads to membrane stabilization, atrioventricular block, QRS interval widening and QT interval lengthening, which leads to serious heart diseases such as conduction disorder, but it is rare	Monitor electrocardiogram	(43–48)
	LEF	It can relieve cardiac hypertrophy induced by pressure overload or angiotensin II *in vivo* and prevent the induction of cardiac fibrosis However, leflunomide has the risk of raising blood pressure	Monitor blood pressure	(52, 53)
	CsA	The decrease of intima-media thickness and plaque prevalence can prevent myocardial hypertrophy, but it can cause blood pressure increase and microvascular injury.	Monitor blood pressure	(32, 57–61)
	AZA	By downregulating TLR4 signaling pathway, it can inhibit the excessive oxidative stress and inflammatory reaction caused by myocardial ischemia and reperfusion, promote the balance of oxidation and antioxidation, and alleviate the process of myocardial injury caused by ischemia and reperfusion to a certain extent	–	(63)
bDMARDs:	TNF antagonists	Improve insulin sensitivity and endothelial function to reduce the risk of cardiovascular disease But it is not recommended for patients with heart failure	Use with caution in patients with heart failure	(67–79)
	Rituximab	By exhausting B cells, the infarct area is reduced and cardiac remodeling is improved, and a few patients may have peripheral edema, hypertension, hypotension or arrhythmia, and myocardial infarction.	Monitor blood pressure and electrocardiogram	(80, 81)
	Abatacept	Improve endothelial function and insulin resistance to reduce the risk of cardiovascular disease	–	(83–87)
	Tocilizumab	Inhibit the migration of human aortic smooth muscle cells and play an anti-atherosclerosis role	–	(91)
	Anakinra	It can reduce heart remodeling after myocardial infarction without increasing the risk of heart failure, and can restore left ventricular diastolic function for patients with existing heart failure, especially patients with diastolic heart failure	Anakinra is suitable for patients with myocardial infarction	(92–96)
tsDMARDs	JAK inhibitors	Increased risk of venous thromboembolic events	Use with caution if there are high-risk factors for venous thromboembolism	(97–99)
Botanic drug	Tripterygium wilfordii	It has protective effect on kidney damage and myocardial ischemia-reperfusion injury caused by salt-sensitive hypertension in rats It can cause cardiac toxicity, and its clinical manifestations are chest tightness, palpitation, arrhythmia, etc. In severe cases, it may cause insufficient blood supply to the heart, sudden drop in blood pressure, shock or heart failure, which is life-threatening	Recommended for RA patients with angina pectoris and myocardial infarction. Monitor blood pressure and electrocardiogram	(102–105)
	TGP	It can ameliorate hypertension and hyperlipidemia, inhibit the progression of atherosclerosis, and protect myocardial ischemia-reperfusion injury.	especially suitable for patients with myocardial infarction or insulin resistance.	(110–114)

## Conclusion

Different anti-rheumatism drugs have different effects on the cardiovascular risk of patients with RA, and their advantages and disadvantages vary. The advantages and disadvantages of different drugs and individualized treatment plans suitable for patients according to the optimal treatment principle should be weighed according to the specific conditions of the patients when choosing an appropriate anti-rheumatism treatment.

## Author Contributions

MD designed and supervised the study. YB reviewed the literature, wrote the original manuscript, and revised the manuscript. ZXi, WY, and ZXu helped with the literature search. MD, ZL, and XK modified the article. All authors contributed to the article and approved the submitted version.

## Funding

This project was supported by the National Natural Science Foundation of China (Grant No. 81771768 and 82001741).

## Conflict of Interest

The authors declare that the research was conducted in the absence of any commercial or financial relationships that could be construed as a potential conflict of interest.

## Publisher's Note

All claims expressed in this article are solely those of the authors and do not necessarily represent those of their affiliated organizations, or those of the publisher, the editors and the reviewers. Any product that may be evaluated in this article, or claim that may be made by its manufacturer, is not guaranteed or endorsed by the publisher.
